# Blood Cell Profiles of the Tadpoles of the Dubois's Tree Frog, *Polypedates teraiensis* Dubois, 1986 (Anura: Rhacophoridae)

**DOI:** 10.1100/2012/701746

**Published:** 2012-05-02

**Authors:** Madhusmita Das, Pravati Kumari Mahapatra

**Affiliations:** Cell and Developmental Biology Laboratory, P.G. Department of Zoology, Utkal University, Bhubaneswar-751 004, Odisha, India

## Abstract

The present paper describes a sequential study of the leukocyte profiles and the changes in morphometry and morphology of erythrocytes in the tadpoles of *Polypedates teraiensis* during their development and metamorphosis, that is, transfer from an aquatic mode to a terrestrial mode of life. Blood smears of 21 different stages (Gosner stage 26 to 46) of tadpoles were investigated. Population of erythrocytes was heterogeneous in population represented by various forms (oval, elliptical or rounded cells, comma shaped, teardrop shaped, schistocytes, senile erythrocytes, crenulated RBCs). Correlation between various morphometric values of erythrocytes was determined with different developing stages of tadpoles. Amongst the leucocytes, the lymphocytes were the most abundant cells followed by neutrophils. Neutrophils and monocytes showed varied morphologic forms. The percentage of lymphocytes and neutrophils showed a negative whereas percentage of eosinophil, basophil, and monocytes showed a positive correlation with the developmental stages of tadpoles. Blood platelets were also observed, which were rounded in shape and found in aggregates.

## 1. Introduction

Anuran metamorphosis is an interesting physiological phenomenon where the aquatic tadpoles become terrestrial froglets. It is a rapid process to meet the demands of a new life form [1]. During early larval development, tissues are formed and body size increases, but later there is a massive tissue restructuring and breakdown leading to a reduction in overall body size [2]. As a part of metamorphosis, blood cell populations are renewed [1], and number of leukocytes changes [2]. Metamorphosis thus provides physiologists an opportunity to understand the processes involved in hematopoiesis. From a developmental and physiological point of view, the changes in the number of leucocytes and the changes in the morphology and morphometry of erythrocytes during the larval development are of great interest and importance. Apart from this, anuran larvae (tadpoles) are study of interest, as they have been widely used as bioindicators to detect the presence of mutagenic agents in water [3–7]. The general nature of blood cell responses to the aquatic environment emphasizes the role of the peripheral blood in tadpoles as a sensitive indicator regarding contamination in aquatic environments [8]. Since anurans are environmentally sensitive animals [8–10] and variation in white blood cell parameters is subjected to environmental stress [11–15], there is a recent interest by ecologists and wildlife researchers in their hematology.

Abundance of different leucocytes under normal metamorphosis [2] and thyroid-induced metamorphosis [16, 17] has been studied in the tadpoles of* Rana catesbeiana*. Granulopoiesis of basophils and heterophils (neutrophils) in the tadpoles of *Rana esculenta* [18] and presence of leucocytes in tissue sections in the tadpoles of *Xenopus laevis* [19] have been reported. Moreover, changes in the number of leucocytes in response to stress in the tadpoles of *Rana esculenta* [8], *Rana catesbeiana* [20], and* Rana pipiens* [21] have been observed.

In larval anurans, two general types of erythrocytes, that is, larval and adult forms, are found, which are differentiated by size and morphology during development [22–24]. The larval form is elongated while the adult form is smaller and rounder [23]. The transition from larval to adult cells has been reported to begin at the onset of metamorphosis in various anuran species like *Rana pipiens* [22] and *Rana catesbeiana* [25–28]. Thyroid-induced destruction of erythrocytes during metamorphosis in the tadpoles of *Hyla crucifer*, *Hyla versicolor*, *Rana clamitans,* and *Bufo americanus* has also been described earlier [29].

Sequential study on the leukocyte profile and changes in the morphometry and morphology of erythrocytes in different larval stages of anurans is not well documented. Moreover, blood cell profile of Indian anurans is restricted to few adult species, namely,* Bufo melanostictus* (new name: *Duttaphrynus melanostictus*) [30–33], *Rana tigerina* (new name: *Hoplobatrachus tigerinus*) [34], and *Polypedates maculatus* [35]. There is no report on the blood cell profiles of tadpoles of any Indian anuran. Thus, aim of the present study was to estimate changes in the leukocyte profile and changes in the morphometry and morphology of the erythrocytes in the laboratory reared anuran tadpoles. This paper describes blood cell profile of the laboratory-reared tadpoles (Gosner stages 26 to 46, [36]) of the Dubois's tree frog* Polypedates teraiensis* (Dubois, 1986) (Anura: Rhacophoridae).

## 2. Material and Methods

### 2.1. Rearing of Tadpoles

 The foam nests of Dubois's tree frog *Polypedates teraiensis* [37] were collected during the month of August 2010 from their natural habitat from Choudwar (20°31′11′′N, 85°49′11′′E), Odisha, India. The tadpoles were reared following standardized procedure [38]. After hatching, the tadpoles were fed with boiled *Amaranthus* leaves *ad libitum*.

### 2.2. Tadpoles Investigated

 For the study, tadpoles from Gosner stages 26 to 46 [36] were considered which were comparable to the Taylor and Kollros stages I to XXV [39, 40]. Ten tadpoles for each stage were selected for investigation.

### 2.3. Preparation of Blood Smears

 Tadpoles were anesthetized by immersing them in 1% MS-222 (Tricaine Methane Sulphonate) solution. The blood of tadpoles from stage 26 to 44 was obtained from tail amputation through the middle of the tail. For stages 45 and 46, blood was collected from the heart using a fine syringe. Blood smears were prepared using push slide technique. The dried blood smears were stained with Giemsa's stain and were observed under light microscope (Hund H500).

### 2.4. Identification and Counting of Blood Cells

 Different types of blood cells present in the smear were identified following Hadji-Azimi et al. [41], Turner [42] and Heatley and Johnson [43]. Slides were viewed in zigzag pattern, covering all parts of the blood smear, and leukocytes were counted in each field of view until 100 cells were counted. 150 fields of view per blood smear were assessed for each stage. Photos of the blood cells were taken with the help of a Canon EOS 450 12.2 Mega pixel camera (EF-S 18-55 1S Kit) connected to Hund H500 WETZLAR microscope. The sizes of red blood cells (RBCs) and their nuclei were measured by an ocular micrometer that was standardized against a stage micrometer (ERMA, Japan made). For morphometry of the erythrocytes, the formulae described by Arserim and Mermer [44] were followed.

### 2.5. Statistical Analysis

The relationship between developmental stages and blood cell profile was assessed by drawing scatter plots. The correlation coefficient “*r*” was calculated in each case by Karl Pearson's method [45].

## 3. Results

A cumulative account of the blood cell profiles of tadpoles of *Polypedates teraiensis *from Gosner stages 26 to 46 is represented in Tables 1 and 2.

### 3.1. Morphology of Blood Cells

#### 3.1.1. Erythrocytes

**Figure 1:**
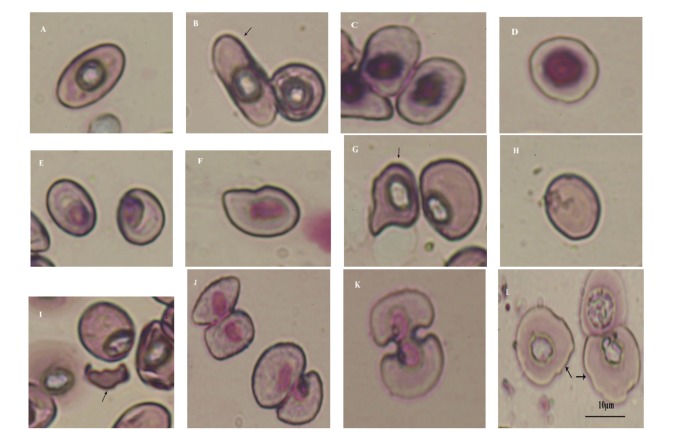
Erythrocytes of the tadpoles of *Polypedates teraiensis*. (A) Oval cell with centrally placed nucleus, (B) and (C) elliptical cells with centrally placed nuclei, (D) round cell with centrally placed nucleus, (E) oval cells with eccentrically placed nuclei, (F) comma-shaped cell, (G) tear-drop-shaped cell, (H) senile erythrocytes (without nucleus), (I) schistocytes (fragmented erythrocytes), (J) and (K) dividing erythrocytes, and (L) large erythrocytes (scale bar = 10 *μ*m).

**Figure 2:**
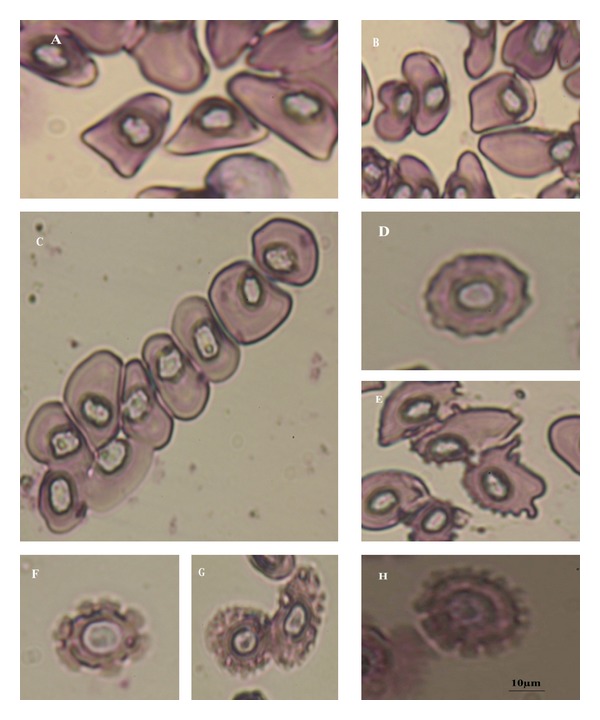
(A) and (B) Poikilocytosis, (C) aggregation of erythrocytes or rouleaux formation, (D) crenulated erythrocytes (resembling echinocytes), (E) crenulated erythrocytes (resembling acanthocytes), (F), (G), and (H) destruction of erythrocyte membrane (scale bar = 10 *μ*m).

The erythrocytes were observed to occur in various forms. They were either elongated or circular (round) in shape. Elongated cells were oval (cells with perfect and smooth curves at both the ends) (Figure 1(A)) and elliptical (cells with more of a rectangular look) (Figures 1(B) and 1(C)). Oval (Figure 1(A)), elliptical (Figures 1(B) and 1(C)), and circular (Figure 1(D)) erythrocytes were observed in all stages. Nuclei in the erythrocytes were either centrally (Figures 1(A), 1(B), 1(C), and 1(D)) or eccentrically (Figure 1(E)) placed. Besides, some irregular forms such as comma-shaped (Figure 1(F)) and dacrocyte, that is, teardrop-shaped (Figure 1(G)) erythrocytes were observed in tadpoles of stages 37 to 40. Senile erythrocytes (without nucleus) and schistocytes (erythrocyte fragments) were observed in the tadpoles of stages 41 and 42 (Figures 1(H) and 1(I)). Dividing RBCs (Figures 1(J) and 1(K)) were present in all the metamorphic stages (39 to 44). In stages 42 to 45, larger erythrocytes were observed (Figure 1(L)). However, poikilocytosis was seen in the early stages from stage 26 to stage 31 (Figures 2(A) and 2(B)). Aggregation of the erythrocyte (Figure 2(C)) or rouleaux formation was observed in almost all stages of tadpoles investigated. During the metamorphic stages (stages 41 to 45), crenulation (short blunt projections or throne-like spicules of variable size distributed over the surface of the cell) of the erythrocytes (Figures 2(D) and 2(E)) and destruction of erythrocyte membranes (Figures 2(F), 2(G), and 2(H)) was distinct.

#### 3.1.2. Leucocytes

**Figure 3:**
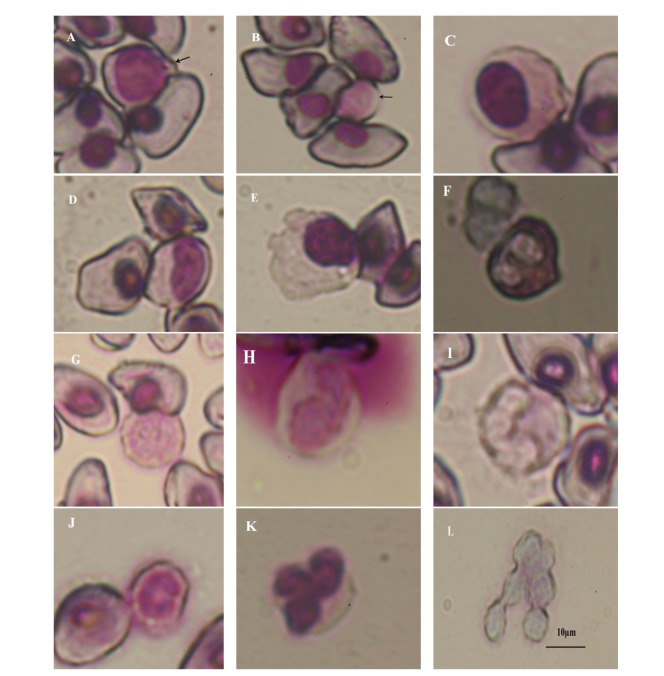
Leucocytes and platelets of the tadpoles of *Polypedates teraiensis. *(A) Large lymphocyte, (B) small lymphocyte, (C) monocyte with eccentrically placed round nucleus, (D) monocyte with intended nucleus, (E) monocyte with pseudopodia-like cytoplasmic extensions, (F) eosinophil, (G) basophil (H) neutrophil with bilobed nucleus, (I) neutrophil with eccentrically placed trilobed nucleus, (J) neutrophil with U-shaped nucleus (band neutrophil), (K) neutrophil with distinct trilobed nucleus, and (L) round platelets in cluster (scale bar = 10 *μ*m).

The leucocytes observed in the present study were of five types, that is, lymphocytes (large and small), monocytes, eosinophils, basophils, and neutrophils. The first two being agranulocytes and the rest were granulocytes. Most of the lymphocytes were rounded in shape. The nuclei were rounded in shape both in large (Figure 3(A)) and small lymphocytes (Figure 3(B)). In both the cells, the nuclei occupied the entire cell leaving a narrow rim of light violet cytoplasm towards the periphery. Monocytes were rounded in shape with either round-shaped nuclei (Figure 3(C)) or indented nuclei (Figure 3(D)). A few monocytes had pseudopod-like cytoplasmic extensions (Figure 3(E)). Eosinophils had bilobed nuclei, which were located at one end of cells (Figure 3(F)). Basophils were round cells having large dark violet-stained granules over the irregular nuclei and also entire cells (Figure 3(G)). Neutrophils showed bilobed, trilobed, or U-shaped nuclei (band neutrophils) (Figures 3(H), 3(I), 3(J), and 3(K)).

#### 3.1.3. Platelets

Blood platelets were rounded in shape and found in clusters (Figure 3(l)).

### 3.2. Morphometry of Erythrocytes

The length and breadth of red blood cells ranged from 19.24 ± 1.41 *μ*m (Gosner stage 35) to 24.24 ± 2.14 *μ*m (Gosner stage 42) and 15.15 ± 0 *μ*m (Gosner stage 30) to 22.14 ± 1.61 *μ*m (Gosner stage 27), respectively (Table 1). Similarly, the length and breadth of the nuclei ranged from 9.09 ± 0 *μ*m (Gosner stage 30) to 12.12 ± 1.11 *μ*m (Gosner stage 27) and 9.09 ± 0 *μ*m (Gosner stage 30, 32, 42 to 46) to 12.12 ± 2.14 *μ*m (Gosner stage 31), respectively, in different developmental stages (Table 1). Moreover, area occupied by the red blood cells ranged from 252.321 ± 31.41 *μ*m^2^ (Gosner stage 36) to 403.908 ± 43.16 *μ*m^2^ (Gosner stage 27), while the nuclear area ranged from 64.720 ± 0 *μ*m^2^ (Gosner stage 30) to 168.290 ± 18.12 *μ*m^2^ (Gosner stage 28) in the tadpoles of different developmental stages (Table 1).

### 3.3. Differential Leukocyte Count

Lymphocytes were the most abundant cells amongst the leucocytes (Table 2). Percentage of lymphocytes ranged from 55.2 ± 1.5 (39) to  63 ± 0.89  (stage 32). Neutrophils were the second abundant cells after lymphocytes. The percentage of neutrophils ranged from  9 ± 2.5  (stage 40) to 32.5 ± 0.9 (stages 26). There was an abrupt decline in the percentage of neutrophils from stage 39 to stage 40, that is,  18 ± 0.55  to  9 ± 2.5  percent, which further increased and fluctuated between 17 to 20 within stages 41 to 46. The neutrophil percentage was observed to be high during the early larval period (stage 26 to 34). The percentage of monocytes ranged from 0 to  12 ± 0.11, and interestingly the maximum percentage was observed at stage 40. At stages 27 and 28, no monocytes were observed whereas the percentage remained higher in stages 40 to 43 (10–12). The percentage of eosinophils ranged from 2.7 ± 0.1 (stage 30) to 15.9 ± 1.12 (stage 40). Tadpoles from stage 34 to stage 42 showed a comparatively higher percentage of eosinophils (9.5 ± 0.25 to 15.9 ± 1.12). Basophil percentage ranged from 1 to 7.9 ± 0.75 with a minimum at stage 26 and maximum at stage 33.

### 3.4. Statistical Analysis

**Figure 4:**
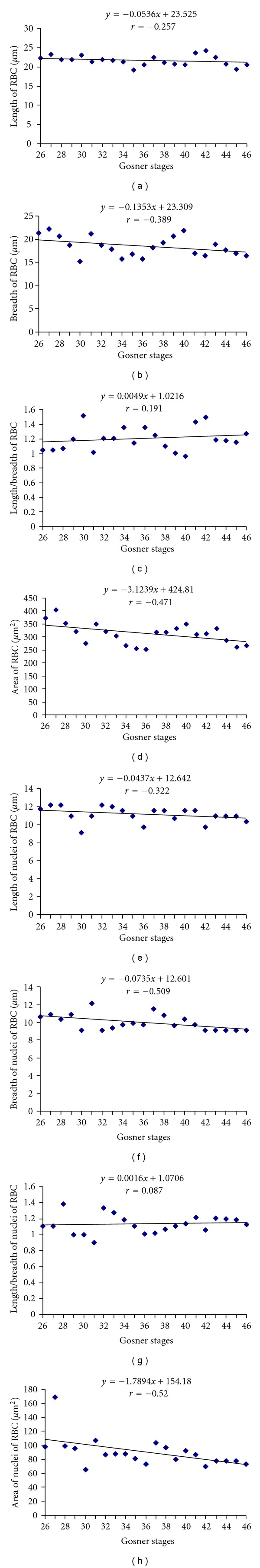
Correlation between different morphometric values of erythrocyte and stages of tadpoles.

A negative correlation was observed between different stages of tadpoles with respect to length (*r* = −0.257, *P* = 0.137), breadth (*r* = −0.389, *P* = 0.125), and area (*r* = −0.471, *P* = 0.114) of the erythrocytes. Similar negative correlation was observed for nuclear length (*r* = −0.322, *P* = 0.131), breadth (*r* = −0.509, *P* = 0.109), and area (−0.520, *P* = 0.107). However, the aspect ratio (length/breadth) of erythrocytes was positively correlated both for cells (*r* = 0.191, *P* = 0.141) as well as nuclei (*r* = 0.087, *P* = 0.146) (Figures 4(a)–4(h)). 

**Figure 5:**
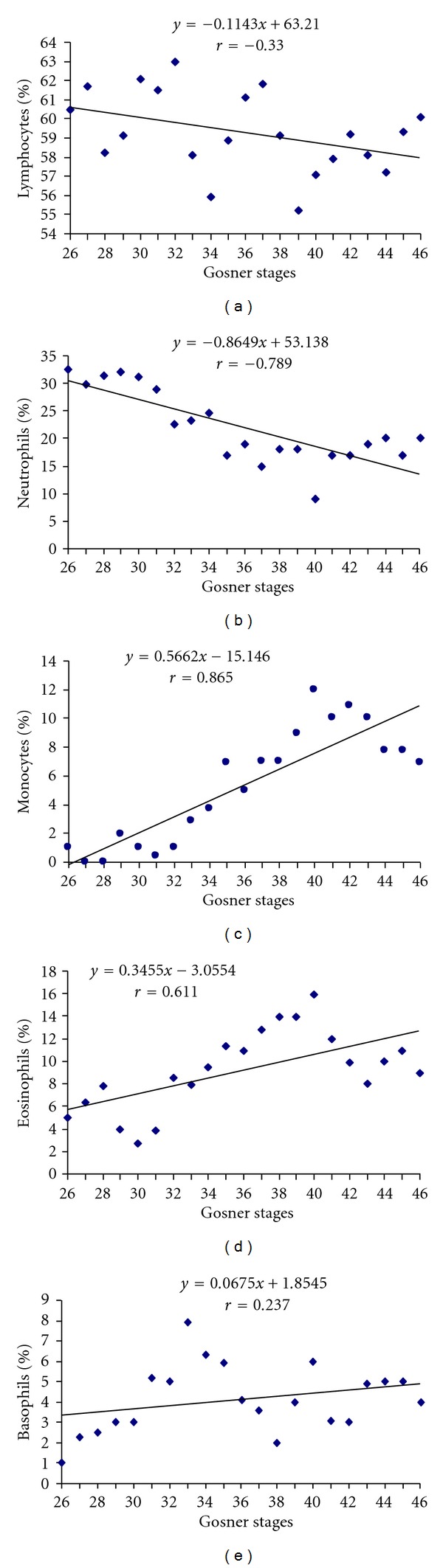
Correlation between percentage of different leucocytes and stages of tadpoles.

 The monocytes (*r* = 0.865, *P* = 0.03), basophils (*r* = 0.237, *P* = 0.13), and eosinophils (*r* = 0.6111, *P* = 0.09) showed a positive correlation, whereas neutrophil (*r* = −0.789, *P* = 0.05) and lymphocytes (*r* = −0.330, *P* = 0.13) showed a negative correlation with different stages during development (Figures 5(a)–5(e)). The percentage of neutrophils, eosinophils, and monocytes showed a significant correlation with different stages during development.

## 4. Discussion

Present investigation describes changes in the shape, size, and number of blood cells during development and metamorphosis in the tadpoles of *Polypedates teraiensis*. The erythrocytes constituted an entirely heterogeneous population. They appeared in different shapes such as elliptical, oval, and rounded. Depending on the position of the nuclei in the cells, it was observed that there existed oval or elliptical cells with nuclei of varying degree of eccentricity (Figure 1(E)), oval or elliptical cells with centrally placed nuclei (Figures 1(A), 1(B), and 1(C)) or rounded cells with centrally placed nuclei (Figure 1(D)). 

Poikilocytosis of RBC with irregular shapes was observed in patches only during early stages, that is, 26 to 31 (Figures 2(A) and 2(B)). Disappearance of these cells at later stage seems to be a normal phenomenon. Another interesting observation was aggregation of erythrocytes or rouleaux formation (Figure 2(C)). In contrast to poikilocytosis, rouleaux formation occurred throughout the larval period. Abnormal shapes of erythrocytes such as teardrop (Figure 1(F)) and comma shaped (Figure 1(E)) were observed in the tadpoles of stages 37 to 40. Apart from this, cells with crenulations (projections over surface of cell) were seen (Figures 2(D) and 2(E)), in stages 41 to 45, which resembled the echinocytes and acanthocytes seen in mammals [46]. Similar, crenulations in red blood cells have been reported in different ranid frogs studied by Hollyfield [22]. He has described small crenulated erythrocytes to appear in the circulation of *Rana pipiens* during metamorphosis, which increases in number as metamorphosis proceeds, and gradually lose their wrinkled appearance. At the end of metamorphosis, the entire red cell population is replaced by these new cells. Vankin et al. [47] have observed cell outlines to be more irregular and crenulated with many cytoplasmic projections in thyroid-treated tadpoles of *R. catesbeiana *and have correlated it with anemic conditions and death of tadpoles during metamorphosis. In vertebrates, the abnormal cells (echinocytes, acanthocytes, schistocytes, teardrop cells, and comma shaped cells) have been reported to be present during anemic conditions [48], and the ectothermic animals are capable of withstanding the anemic conditions for a long period without mortality [49]. Thus, the present findings indicate the tadpoles to pass through a critical condition where erythrocytes show variations in shapes. 

Senile erythrocytes (without nuclei) and schistocytes (erythrocytes fragments) were common in stages 41 and 42. Presence of the senile erythrocytes has been reported during metamorphosis in tadpoles of *Hyla crucifer, Hyla versicolor, Rana clamitans, and Bufo americanus* [29]. Schistocytes described in the present study may be due to the fragmentation of the erythrocytes as a result of passage through the blood vessels due to change in the metabolic activities during metamorphosis at stages 41 and 42. 

Apart from this, division of erythrocytes was also observed from stages 39 to 44. This may be due to increase in erythropoietic activity during metamorphosis as reported earlier by Maniatis and Ingram [50] in *Rana catesbeiana*. Comparatively larger erythrocytes observed in stages 42 to 45 of the present study resembled the immature erythrocytes described in the tadpoles of *Rana catesbeiana *[50]. 

Size (length and breadth) of erythrocytes and their nuclei was observed to be negatively correlated with the developmental stages of the tadpoles (Figures 4(a), 4(b), 4(e), and 4(f)). The aspect ratio (length/breadth) of both the erythrocytes and their nuclei showed a positive correlation (Figures 4(c) and 4(g)), which indicated the formation of rounded cells with the growth of tadpoles. Several other workers have reported that during anuran metamorphosis larger larval cells were replaced by smaller adult erythrocytes [51, 52]. In the present study, the area of erythrocytes decreased with the development of the tadpoles (Figure 4(d)). Similar decrease in the erythrocyte area has been reported in tadpoles of *Rana catesbeiana* [22, 23, 28]. Decrease in area of erythrocytes during metamorphosis seems to be associated with the transfer from an aquatic to a terrestrial mode of life of the tadpole larva. 

The leukocyte population (the nonspecific immune system) is reported to be one of the primary lines of defense against invading pathogens and is made up of five different types of white blood cells, each performing different tasks in the immune process [53, 54] in anurans. Out of the five types of leucocytes studied, the lymphocytes showed a negative correlation with the developmental stages (Figure 5(a)). Davis [2] has reported higher percentage of lymphocytes (70%) in the tadpoles of stages 30 to 33 in *Rana catesbeiana*, which declined with the onset of metamorphosis. Neutrophils also showed a negative correlation with the developmental stages (Figure 5(b)). Since, neutrophils are regarded as the first cell to respond to the site of infections [55], presence of a higher percentage of neutrophil in the early developmental stages represents requirement of more nonspecific immunity. Similar observation is seen in tadpoles of *Rana catesbeiana* [2]. A positive correlation was observed between the monocyte percentage and developmental stages. Monocyte percentage remained higher from stage 40 to stage 43, suggesting their involvement in the early stages of metamorphosis. Elevation in the percentage of monocytes is suggested to be correlated with increase in cellular debris resulted during remodeling of the larval structures. The percentage of eosinophils was positively correlated with progress in development. Eosinophils are known to produce a number of chemicals to initiate and modulate the immune and inflammation response [56, 57]. Thus, eosinophils may act to modulate the process of lysis of tissue during metamorphosis, which behave in a way similar to the inflammation response [2]. Basophils showed a positive correlation (Figure 5(e)), which was not significant. In the tadpoles of *Rana catesbeiana* [2], general increase in the number of basophils has been reported, and the author suggested the trend to be related to their formation and entrance in circulation rather than a direct association with metamorphosis. 

Thus, the present study provides information regarding the blood cells of the tadpoles of *Polypedates teraiensis* during their development and metamorphosis, that is, their transfer from an aquatic to a terrestrial mode of life. As this is the first study to examine the hematological values of the tadpoles of *Polypedates teraiensis*, it establishes a baseline data that can be used as general reference values for future investigations involving this species and other anuran tadpoles. 

## Figures and Tables

**Table 1:** Morphometry of erythrocytes during development of the tadpoles of *Polypedates teraiensis*.

Stages		Size of cell				Size of nuclei				
Gosner	Taylor and Kollros	Length (L ± SD) (*μ*m)	Breadth (B ± SD) (*μ*m)	L/B ± SD	Area (A ± SD) (*μ*m²)	Length (L′ ± SD) (*μ*m)	Breadth (B′± SD) (*μ*m)	L′/B′± SD	Area (A′± SD) (*μ*m²)	A′/A ± SD
26	I	22.22 ± 1.26	21.30 ± 2.11	1.043 ± 0.14	371.529 ± 40.61	11.69 ± 1.51	10.60 ± 1.32	1.102 ± 0.12	97.373 ± 12.39	0.261 ± 0.12
27	II	23.24 ± 1.31	22.14 ± 1.61	1.049 ± 0.16	403.908 ± 43.16	12.12 ± 1.11	10.90 ± 1.41	1.111 ± 0.11	168.290 ± 18.12	0.416 ± 0.03
28	III	21.81 ± 1.35	20.60 ± 2.53	1.066 ± 0.12	353.254 ± 50.47	12.12 ± 1.91	10.30 ± 1.48	1.382 ± 0.17	99.488 ± 27.51	0.390 ± 1.41
29	IV	21.81 ± 2.53	18.78 ± 3.95	1.20 ± 0.301	321.549 ± 80.77	10.90 ± 1.65	10.90 ± 1.65	1 ± 0	95.075 ± 27.71	0.306 ± 0.11
30	V	23.02 ± 2.71	15.15 ± 0	1.52 ± 0.17	274.005 ± 32.24	9.09 ± 0	9.09 ± 0	1 ± 0	64.720 ± 0	0.239 ± 0.033
31	VI	21.21 ± 2.14	21.21 ± 2.14	1.016 ± 0.206	350.130 ± 30.66	10.90 ± 2.71	12.12 ± 2.14	0.9 ± 0.13	106.605 ± 44.76	0.247 ± 0.17
32	VII	21.81 ± 2.53	18.78 ± 4.97	1.21 ± 0.30	321.596 ± 87.66	12.12 ± 2.14	9.09 ± 0	1.33 ± 0.23	86.521 ± 15.30	0.276 ± 0.06
33	VIII	21.61 ± 2.16	17.87 ± 1.1	1.209 ± 0.21	303.143 ± 32.12	11.96 ± 1.26	9.39 ± 1.12	1.273 ± 0.21	88.158 ± 11.62	0.290 ± 0.02
34	IX	21.21 ± 2.14	15.75 ± 2.53	1.35 ± 0.12	265.352 ± 64.77	11.51 ± 1.35	9.69 ± 1.35	1.19 ± 0.18	87.951 ± 17.94	0.350 ± 0.11
35	X	19.24 ± 1.41	16.87 ± 1.21	1.140 ± 0.11	254.794 ± 41.12	10.96 ± 1.35	9.93 ± 1.21	1.103 ± 0.17	80.787 ± 12.91	0.317 ± 0.05
36	XI	20.60 ± 2.53	15.75 ± 2.53	1.35 ± 0.34	252.321 ± 31.41	9.69 ± 1.35	9.69 ± 1.35	1.01 ± 0.20	73.529 ± 11.86	0.293 ± 0.05
37	XII	22.42 ± 2.71	18.18 ± 2.14	1.248 ± 0.22	318.711 ± 41.54	11.51 ± 1.35	11.51 ± 1.35	1.016 ± 0.20	103.798 ± 15.76	0.326 ± 0.04
38	XIII	21.12 ± 1.31	19.20 ± 1.2	1.10 ± 0.22	318.320 ± 32.61	11.51 ± 1.61	10.75 ± 1.12	1.070 ± 0.21	97.130 ± 11.12	0.305 ± 0.04
39	XIV	20.63 ± 1.41	20.60 ± 1.61	1.00 ± 0.11	333.607 ± 31.41	10.64 ± 1.11	9.63 ± 1.11	1.104 ± 0.25	80.4333 ± 9.31	0.241 ± 0.06
40	XV-XVII	20.60 ± 2.53	21.81 ± 2.53	0.96 ± 0.22	350.336 ± 35.89	11.51 ± 1.35	10.30 ± 1.65	1.14 ± 0.26	92.284 ± 12.87	0.267 ± 0.06
41	XVIII-XIX	23.63 ± 3.95	16.96 ± 2.71	1.43 ± 0.39	310.058 ± 35.69	11.51 ± 1.35	9.69 ± 1.35	1.21 ± 0.25	86.528 ± 0	0.282 ± 0.03
42	XX	24.24 ± 2.14	16.36 ± 1.65	1.49 ± 0.20	311.500 ± 40.59	9.69 ± 1.35	9.09 ± 0	1.06 ± 0.14	69.196 ± 9.68	0.227 ± 0.06
43	XXI	22.42 ± 2.11	18.93 ± 0.21	1.184 ± 0.21	333.162 ± 30.69	10.93 ± 1.32	9.09 ± 0	1.202 ± 0.12	77.992 ± 9.29	0.234 ± 0.05
44	XXII	20.64 ± 2.12	17.63 ± 0.69	1.170 ± 0.20	285.648 ± 31.41	10.90 ± 1.51	9.09 ± 0	1.199 ± 0.14	77.778 ± 9.48	0.272 ± 0.06
45	XXIII-XXIV	19.39 ± 3.45	16.96 ± 2.71	1.15 ± 0.22	259.565 ± 66.31	10.90 ± 1.65	9.09 ± 0	1.19 ± 0.18	77.862 ± 11.86	0.304 ± 0.04
46	XXV	20.60 ± 2.53	16.36 ± 2.71	1.27 ± 0.19	266.768 ± 64.49	10.30 ± 1.65	9.09 ± 0	1.13 ± 0.18	73.529 ± 11.86	0.280 ± 0.05

Correlation coefficient (*r*)		−0.257	−0.389	0.191	−0.471	−0.322	−0.509	0.087	−0.520	−0.305

SD: standard deviation.

**Table 2:** Percentage of leucocytes throughout larval development in *Polypedates teraiensis*.

Stages		Lymphocytes ± SD	Neutrophils ± SD	Monocytes ± SD	Eosinophils ± SD	Basophils ± SD
Gosner stage	Taylor and Kollros stages					
26	I	60.5 ± 1.2	32.5 ± 0.9	1 ± 0	5 ± 0.1	1 ± 0.0
27	II	61.7 ± 1.1	29.7 ± 0.9	0 ± 0	6.3 ± 0.12	2.3 ± 0.11
28	III	58.2 ± 1.01	31.5 ± 0.85	0 ± 0	7.8 ± 0.12	2.5 ± 0.11
29	IV	59.1 ± 0.9	32 ± 0.75	1.9 ± 0.1	4 ± 0.21	3 ± 0.12
30	V	62.1 ± 0.98	31.2 ± 0.75	1 ± 0	2.7 ± 0.1	3 ± 0.12
31	VI	61.5 ± 0.9	29 ± 1.75	0.4 ± 0	3.9 ± 0.2	5.2 ± 0.21
32	VII	63 ± 0.89	22.5 ± 0.75	1 ± 0	8.5 ± 0.55	5 ± 0.21
33	VIII	58.1 ± 0.89	23.2 ± 0.75	2.9 ± 0.1	7.9 ± 0.12	7.9 ± 0.75
34	IX	55.9 ± 0.97	24.6 ± 0.85	3.7 ± 0.11	9.5 ± 0.25	6.3 ± 0.75
35	X	58.9 ± 1.01	17 ± 1.2	6.9 ± 0.21	11.3 ± 0.55	5.9 ± 0.25
36	XI	61.1 ± 1.2	18.9 ± 1.1	5 ± 0.11	10.9 ± 0.25	4.1 ± 0.25
37	XII	61.8 ± 1.2	14.8 ± 0.75	7 ± 0.12	12.8 ± 0.26	3.6 ± 0.12
38	XIII	59.1 ± 1.3	18 ± 0.75	7 ± 0.12	13.9 ± 0.35	2 ± 0.11
39	XIV	55.2 ± 1.5	18 ± 0.55	8.9 ± 0.12	13.9 ± 0.35	4 ± 0.25
40	XV-XVII	57.1 ± 1.5	9 ± 2.5	12 ± 0.11	15.9 ± 1.12	6 ± 0.75
41	XVIII-XIX	57.9 ± 1.1	17 ± 1.20	10 ± 0.13	12 ± 1.10	3.1 ± 0.12
42	XX	59.2 ± 0.95	17 ± 1.25	10.9 ± 0.11	9.9 ± 0.75	3 ± 0.12
43	XXI	58.1 ± 0.95	19 ± 1.5	10 ± 0.11	8 ± 0.75	4.9 ± 0.25
44	XXII	57.2 ± 0.75	20 ± 1.25	7.8 ± 0.12	10 ± 0.55	5 ± 0.25
45	XXIII-XXIV	59.3 ± 1.25	17 ± 1.25	7.8 ± 0.12	10.9 ± 0.75	5 ± 0.25
46	XXV	60.1 ± 1.10	20 ± 1.25	6.9 ± 0.2	9 ± 0.75	4 ± 0.55

Correlation coefficient (*r*)		−0.330	−0.789	0.865	0.611	0.237

SD: standard deviation.
